# Quest for financial inclusion via digital financial services (Fintech) during COVID-19 pandemic: case study of women in Indonesia

**DOI:** 10.1057/s41264-023-00217-9

**Published:** 2023-03-13

**Authors:** Budi Setiawan, Thich Dai Phan, Jennifer Medina, Martijn Wieriks, Robert Jeyakumar Nathan, Maria Fekete-Farkas

**Affiliations:** 1 Doctoral School of Economic and Regional Studies, Hungarian University of Agriculture and Life Sciences, Gödöllő, Hungary; 2 Faculty of Economics, Universitas Indo Global Mandiri, Palembang, Indonesia; 3 Faculty of Economics, University of Miskolc, Miskolc, Hungary; 4 Faculty of Economics and Business Administration, University of Seville, Seville, Spain; 5 Chief Data Officer, JULO, South Jakarta, Indonesia; 6 Faculty of Business, Multimedia University, Cyberjaya, Malaysia; 7 Faculty of Economics, University of South Bohemia in České Budějovice, České Budějovice, Czech Republic

**Keywords:** Financial inclusion, Financial technology, Indonesian gender gap, TAM, Women in Fintech, UN SDG8, G23, J16, O33

## Abstract

Based upon an extended Technology Acceptance Model (TAM), this study aims to investigate the factors influencing the behavioral intention to adopt Fintech from the perspective of Indonesian women. The research data were collected from 409 Indonesian female respondents and analyzed using the SEMinR statistical data analysis tool. Structural equation modeling (SEM) was used to assess this research’s measurement model and structural model. The result shows that perceived usefulness, perceived ease of use, user innovativeness, attitude, trust, and brand image significantly positively impact behavioral intention to adopt Fintech among Indonesian women. Meanwhile, perceived ease of use, financial literacy, and government support are found to have indirect relationships with behavioral intention. In addition, moderation analysis revealed that the saving habits of women during the COVID-19 pandemic reduced the relationship between their innovativeness and behavioral intention to adopt Fintech. Based on these results, we recommend practical suggestions to the government, policymakers, and aspiring Fintech service providers further to enhance women’s empowerment through digital financial inclusion.

## Introduction

The World Bank (2017) reveals that the world’s unbanked population has decreased from 2 billion in 2014 to approximately 1.7 billion in 2017. However, improving access to financial services still faces numerous obstacles, most notably the gender disparity in financial access. Women’s participation in financial services is unequal to men from all levels, such as depositors, borrowers, managers, and regulators (Sahay and Cihak 2018). Demirgüc-Kunt et al. (2018) found that 72% of men have access compared to 65% of women globally. Furthermore, in developing nations, the gender gap in financial access is widening; women have 8% less access to financial services than men (Demirgüc-Kunt et al. 2018). Financial and non-financial variables contribute to the gender gap in accessing financial services. The high cost of opening and maintaining the account is the financial factor for low financial inclusion (Allen et al. 2012). Women face non-financial barriers to financial inclusion, ranging from insufficient documents to subjective variables such as culture and mindset (Hammer and Dahan 2015; Khera et al. 2022). In addition, numerous nations’ social norms and discriminatory regulations have restricted women’s financial participation (Torontocentre 2019; World Bank 2018).

The arrival of financial technology (Fintech) has the potential to close the gender gap in women’s access to financial services (Guo et al. 2021) by offering substantially lower costs than traditional financial business and making financial products accessible to all segments of society (Demirgüc-Kunt and Klapper 2013). In addition, the technological advancement of Fintech empowers the optimization of consumer data analysis and influences the credit-scoring system (Mialou et al. 2017; Gomber et al. 2018). Fintech also provides solutions to consumer choices, avoids expenses unrelated to consumer financial needs (Xu and Zia 2012), satisfies specific consumer needs (niche market), and enables acquiring new clients via cross-selling strategy (Feyen et al. 2021).

Despite the rapid growth of smartphone users and internet penetration in Indonesia, the country’s Fintech ecosystem lags behind its neighbors (Zheng et al. 2022). The number of Fintech companies in Indonesia is 352 as of May 2022, compared to 6 companies in 2006 (Developersbri 2022). More than half of Fintech companies in Indonesia offer digital payment systems and peer-to-peer lending (P2P lending) services. Other Fintech businesses are focused on crowdfunding, microfinance, and e-aggregator. In 2021, Fintech loan disbursements reached IDR 295.85 trillion, an increase of 89 percent over the previous year (OJK 2022). The Indonesian government also encourages the growth of the Fintech companies by involving them in the national economic recovery program to distribute direct cash funding as part of the social safety net during the COVID-19 outbreak (OJK 2020; Sugandi 2021). Despite the growth of Indonesia’s Fintech industry, the challenges of providing formal financial access to all levels continue. World Bank (2017) reported that 95 million Indonesian people remained unbanked in 2017. The financial access barriers in Indonesia are caused by infrastructural, cost, and geographical. Other aspects, including regulation and policy, digital user demand, and innovative products, must be enhanced to promote the Fintech ecosystem in Indonesia.

The digital transformation of financial inclusion of women and youth toward financial inclusion is one of the main priorities of Indonesia’s G20 Presidency. The Indonesian government through the Financial Services Authority (Otoritas Jasa Keuangan/OJK) has established the Regional Financial Access Acceleration Team (Tim Percepatan Akses Keuangan Daerah/TPAKD) to promote financial inclusion, including access to financial services for people in rural areas and women, who are considered vulnerable groups affected by the economic crisis and play an important role in household financial decisions (OJK 2018). Furthermore, OJK is also collaborating with the Financial Services Industry (Industri Jasa Keuangan/IJK) initiated the One Student One Account (OSOA) program with a target of achieving 90% of national financial inclusion by 2024. The Indonesian government’s efforts to increase financial inclusion fit into the global financial inclusion agenda, moreover Indonesia is the country with the 4th largest population in the world, and so increasing financial inclusion in Indonesia will contribute to greater financial inclusion in the world.

Several recent empirical studies have assessed the customers’ behavior/satisfaction in adopting or using financial services (Mainardes et al. 2022). Kaur and Arora (2022) found that the impact of effort expectancy and price value on behavior intention toward online banking services is stronger in males than females. In an attempt to investigate the factors in adopting mobile money in Côte d’Ivoire, Nonvide and Alinsato (2022) found that females are more likely to trust this financial service than males. Kim (2022) investigated the relationship between e-money and women’s financial inclusion in Kenya. This study reveals that Fintech has increased women’s access to financial inclusion. However, a more recent study on technology toward financial inclusion conducted by Ghosh (2022a, b) indicated that women are 12 percent less likely than men to utilize a mobile phone while opening accounts. Therefore, with very limited studies, there remains a gap in our understanding of adopting Fintech for women, particularly during the COVID-19 pandemic. Further, previous literature stated that Fintech access had been proven to exhibit saving behaviors (Becker 2017). However, limited evidence of the impact of COVID-19 on saving habits has been highlighted (Gopal and Malliasamy 2022).

Understanding factors that lead to Fintech adoption plays a pivotal role in bridging the gap between women from financial exclusion to inclusion. In light of the paucity of literature, the present study empirically analyzes the influence of some factors on women’s adoption of Fintech during the COVID-19 health crisis. This research’s novelty uses several factors affecting Fintech adoption, such as brand image, financial literacy, and saving habits during the Covid-19 pandemic, and focuses on Indonesian women as respondents. To the authors’ knowledge, this study is the first literature to tap the drivers of Fintech adoption by women in Indonesia and to tackle the timing of the COVID-19 outbreak.

The subsequent section of this study, Sect. "Literature Review", provides theoretical context, prior research, and the methodology. Sect. "Research methodology" summarizes the outcome, which is then discussed in Sect. "Discussions and conclusion" with the main conclusions. Finally, Sect. "Implications, limitations, and future works" adds some implications, recommendations, and limitations.

## Literature review

Theoretically, several works of literature have been connected to Fintech adoption with theories, such as the technology acceptance model (Davis 1985); innovation diffusion theory (Rogers 1995); technology readiness (Parasuraman 2000); united theory of acceptance and use of technology (Venkatesh et al. 2003), united theory of acceptance and use of technology 2 (Venkatesh et al. 2012), and individual innovativeness theory (Roger 2003). This study will focus on the technology acceptance model (TAM) and individual innovativeness theory (IIT). TAM is selected because it is a leading theory for measuring the adoption of new technologies (Shaikh and Karjaluoto 2015). At the same time, Yoon and Lim (2020) argued that individuals’ roles in embracing new technology to boost effectiveness are reflected in IIT.

The facility of access and secure use can increase the formalization of women’s financial transactions, empowering them toward knowledge and control of their financial decisions. Women often face impediments to accessing and using financial services. Murata and Sioson (2018) showed how socio-economic and cultural factors make it difficult for women to access financial institutions. In developing economies, barriers increase for women living in rural areas where access to financial services is difficult, such as distance, family responsibilities, and certain attitudes toward financial institutions. Survase et al. (2021) revealed a gender inequality in digital payment in a study of SAARC countries. Ogawa et al. (2022) explained that the differences in the access and the use of financial services continue existing, but that this gap becomes much larger in the dimension of financial sector leaders (only 20% of board seats are women) and in the dimension of those responsible for financial sector regulations. Guo et al. (2021) found that financial technology reduces the gender wage gap in China by reducing capital constraints and operating costs, thus promoting female entrepreneurship, driving more women to work, and allowing them to increase their wages. Sahay et al. (2020) documented that financial technologies play a positive role in closing the gender gap in some developing regions and show that the gap tends to be larger or smaller for Fintech-driven inclusion than for traditional financial inclusion, depending on the regions, which can be explained by culture and barriers.

Using a database published by the World Bank, Sioson and Kim (2019) reflected a gender gap even when the percentage of female account holders in Asia–Pacific has increased in recent years and show differences in the gap in the comparative between countries. However, Singh et al. (2020) studied the influence of gender on the antecedents of Fintech use and indicate that gender does not alter the dynamics among the attributes of Fintech services by the analysis of a survey where 30% of responses were made by women. Therefore, the geographic setting is important in assessing outcomes due to cultural gender differences. We could also say that the level of comfort with financial transactions varies according to gender. There are empirical works showing that women are more averse to financial risk than men (Fehr-Duda et al. 2006), and single women are even more averse to taking on financial assets than unmarried women (Jianakoplos and Bernasek 1998).

The theory of financial inclusion of vulnerable groups argues that inclusion programs should target vulnerable members of society who suffer the most from economic hardship and crises, such as women, so it may be cost-effective to focus on vulnerable members of the population for financial inclusion rather than generalist policies (Ozili 2020). The correlation between increased financial inclusion and leader decision-making is reflected in the role of persuading policy to encourage accelerated access to financial services. Ghosh (2022a, b) proved that empowering political leaders increases financial service activity. This finding is consistent with the community echelon theory of financial inclusion (Ozili 2020), which considers the important role of communal leaders in influencing their community to encourage access to finance, especially for financially-excluded members.

Empirically, the previous literature on Fintech in Indonesia has focused on broad individual responders, with little mention of women. Nurlaily et al. (2021) analyzed whether there are gender differences in Indonesia in the perceived benefits and risks of using Fintech. Their results revealed that when women perceive more risks using Fintech, they are more likely to abandon use. Haqqi and Suzianti (2020) investigated the benefits and risks of Fintech adoption in Indonesia. The study found that trust, economic benefits, and ease of use influence consumer adoption of Fintech. Recent studies on Fintech acceptance among Indonesian users by Setiawan et al. (2021) indicated that user innovativeness has a direct and indirect impact on Fintech adoption; however, financial literacy does not affect Fintech adoption in Indonesia. Several studies, namely Junger and Mietzner (2020); Morgan and Trinh (2020) and Khera et al. (2022) reveal financial literacy is positively correlated to Fintech adoption.

### Propose hypotheses

The purpose of this study is to investigate the factors leading to the adoption of Fintech for women in Indonesia. Multiple independent variables, including perceived usefulness (PU), perceived ease of use (PEU), user innovativeness (UI), attitude (AT), trust (TR), brand image (BRI), government support (GS), and financial literacy (FL) are observed in this study, and these variables are associated with Fintech adoption through behavior intention (BI). Furthermore, the saving habit is also examined as intervening and moderating variable for the driver factors of Fintech adoption during the COVID-19 pandemic.

### Perceived usefulness (PU)

Davis et al. (1989) define perceived usefulness (PU) as the degree to which technology can contribute to performance improvement. This variable is critical for influencing the continuance of technology adoption (Yan et al. 2021). In this study, PU is determined to measure how Fintech adoption can meet user needs, such as time savings and advantages. Many previous studies have proven a positive correlation between PU and technology adoption (Do and Do 2020; Singh et al. 2020; Talwar et al. 2020; Rahi et al. 2020; Nugraha et al. 2022). However, Mufarih et al. (2020) found that PU is not significant in affecting digital banking adoption. The following hypothesis is offered based on prior research as follows:

#### H1

Perceived usefulness positively impacts behavior intention to adopt Fintech.

### Perceived ease of use (PEU)

The concept of Perceived ease of use (PEU) pertains to an individual’s level of effort required to use new technology (Davis et al. 1989). PEU is defined in this study as the efficiency upon which Fintech services are used, which includes evaluating the Fintech service interface and the simplicity with which Fintech services may be accessed using various electronic devices. The previous study has revealed that PEU has a positive impact on Fintech adoption (Agyei et al. 2020; Chawla and Joshi 2020; Abdul-Halim et al. 2022; Jain and Chowdhary 2021). Based on the above empirical study, the following hypothesis is initiated:

#### H2

Perceived ease of use positively impacts behavior Intention to adopt Fintech.

#### H3

Perceived ease of use has a positive impact on Perceived usefulness.

#### H3m

Perceived usefulness mediates the influence of Perceived ease of use on Behavior intention.

### User innovativeness (UI)

User innovativeness is an attitude that results in producing new ideas (Jahanmir and Cavadas 2018). The study focuses on identifying whether women with innovative behavior influence the adoption of digital financial services. In this study, user innovation is defined as the willingness to explore new technologies, be early adopters of cutting-edge technology, and be eager to experiment with Fintech services. Prior literature has shown that user innovation correlates positively with technology adoption (Setiawan et al. 2021; Twum et al. 2021). Therefore, based on the preceding literature, the proposed hypothesis is as follows:

#### H4

User Innovativeness has a positive impact on behavior intention in adopting Fintech.

### Financial literacy (FL)

Financial literacy is commonly defined as an awareness and understanding of basic finance, which includes financial products, institutions, and concepts; financial skills, such as the ability to calculate compound interest; and, more broadly, financial capability in terms of money management and financial planning (Xu and Zia 2012). This study refers to Lusardi (2019) to measure financial literacy by asking about compound interest, inflation, and risk diversification. Previous research conducted by Varkey (2020), Hasan et al. (2021), Kaiser et al. (2021), and Voros et al. (2021) show a positive correlation between financial literacy and Fintech adoption. Therefore, based on the previous literature, the following hypothesis is advanced to examine the effect of financial literacy on Fintech adoption:

#### H5

Financial literacy has a positive impact on behavior intention to adopt Fintech.

#### H6

Financial literacy has a positive impact on User Innovativeness.

#### H6m

User Innovativeness mediates the influence of Financial Literacy on Behavior intention.

### Government support (GS)

The government supports improving the development of favorable ecosystems for the Fintech sector through the innovation office and a regulatory sandbox is needed (UNSGSA 2019). According to Chinnasamy et al. (2021), government support is the central pillar of Fintech development. Furthermore, various studies demonstrate that government support positively impacts Fintech adoption (Hua and Huang 2021; Kennedyd et al. 2020; Mejia-Escobar et al. 2020). In this study, government support was associated with infrastructure development, legislation, and regulation that promote the Fintech industry’s growth and enhance the development of the network connection. Thus, the following hypothesis is proposed:

#### H7

Government support has a positive impact on behavior intention to adopt Fintech.

#### H8

Government support has a positive impact on User Innovativeness.

#### H8m

User Innovativeness mediates the influence of Government support on Behavior intention.

### Trust (TR)

Trust is the foundation of financial services (Broby 2021). Cojoianu et al. (2021) illustrated that while Trust in incumbent financial services declines in one location, Fintech services develop in the same region. This survey assesses user trust related to personal data protection and security in Fintech services. According to previous studies, Trust positively affects Fintech adoption (Ali et al. 2021; Bin-Nashwan 2020). Hence, concerning previous literature, we hypothesize that:

#### H9

Trust has a positive impact on behavior intention to adopt Fintech.

### Brand image (BRI)

Brand image is critical in building confidence among users of behavior intention in adopting Fintech services. In particular, transactions involving Fintech services are conducted without direct contact between parties. Prior studies on brand image toward Fintech adoption examined many aspects, such as the brand image connected with quality (Riyadh et al. 2010), brand equity (Brexendorf and Keller 2017), and value (Shapiro et al. 2018). This study examines the relationship between brand image and user preferences in adopting Fintech services based on renowned brands, including company reputation. Previous empirical studies indicate that brand image positively impacts behavior intention to adopt Fintech (Caviggioli et al. 2020; Setiawan et al. 2021; Nathan et al. 2022). The hypothesis is proposed for assessing brand image on Fintech adoption based on the above literature:

#### H10

Brand image has a positive impact on behavior intention to adopt Fintech.

### Attitude (AT)

According to Ajzen (1993), attitude is a person’s tendency to evaluate their likes and dislikes toward an object, activity, person, institution, or event. In this study, the attitude was examined by determining if an individual believes adopting Fintech services is a good idea, their level of comfort with the technology, and their level of interest in the service. Previous research that integrated attitude with behavior intention to adopt Fintech was conducted by Akinware and Kyari (2022), Setiawan et al. (2021), and Nathan et al. (2022) revealed attitude significantly correlated with Fintech adoption. Therefore, on the premise of the above empirical study, the following hypothesis is proposed:

#### H11

Attitude has a positive impact on behavior intention to adopt Fintech.

### The moderating effect of saving behavior in the COVID-19 pandemic

Loibl et al. (2011) argued that individual behavior to dedicate a percentage of income continuously to plan for future financing needs or pay debts is a saving habit. During the COVID-19 pandemic, individual perspectives significantly impact financial decisions relating to saving. People who believe that COVID-19 can disrupt supply chains and cause a shortage of food are typically driven to spend more to meet food security in the future (Gomez-Corona et al. 2021). On the other hand, those who feel that COVID-19 would last long without affecting the food supply will likely increase their savings as part of an emergency fund to protect against potential future income declines. In addition, Achtziger et al. (2015) confirmed that self-control is a primary factor for managing expenses, particularly impulse-buying products, the intensity of which is likely to increase along with frequent interactions with Fintech services that facilitate e-commerce transactions. Limiting interactions with Fintech services will reduce user behavior to explore the features available in Fintech mainly to prevent users from making unplanned purchases due to promotions or discounts often offered by Fintech service providers (Sakas et al. 2022). Based on the literature above, the research hypotheses are:

#### H12a, b, c, d, e

The relationship between relevant factors (perceived usefulness, user innovativeness, trust, brand image, attitude) and behavior intention to adopt Fintech is moderated by saving habits in the COVID-19 pandemic.

## Research methodology

### Data collection

This study obtained primary data to answer the research hypotheses. Respondents with basic information about Fintech were qualified to take part in this survey. The original English-language online survey questionnaire was translated into Indonesian by qualified native Indonesian speakers. Data collection through questionnaires was carried out in three stages. First, the researcher developed the questionnaire items based on the theoretical construct (Buschle et al. 2022). Second, a pilot survey was conducted through interviews with respondents. At this stage, respondents were also asked to respond to questions on the questionnaire. Fink (2003) recommended at least 10 samples, and Julious (2005) recommended 12 sample sizes per group for pilot studies. This study collected data from 40 respondents for the pilot survey. The results of the pilot survey analysis were checked for reliability and validity tests and provided insight to make the questionnaire simpler, unambiguous and concise (Saunders et al. 2016). Lastly, the questionnaire that had been corrected at the pilot survey stage was distributed to women respondents in Indonesia to achieve the target sample required in the study. To determine the number of samples in this study, we refer to Sekaran and Bougie (2016), who recommended that sample sizes higher than 30 and lower than 500 are sufficient for most studies. In addition, G* Power software can be applied to calculate the minimum sample size. With a confidence level of 95% at 0.80 power estimates and minimum sample size is 160 (Faul et al. 2009; Jain and Raman 2022). This study collected 426 respondents between 16 January 2022 and 10 May 2022. After eliminating some incomplete data and taking out the respondents’ answers to all questions with the 5 Likert scale, the 409 samples were finalized for analysis.

Table 1 presents that Indonesian young woman respondents between 15 and 25 years old are dominant in age respondent characteristics with almost 45%, compared to people older than 46 who are below 10%. Most Indonesian women in this survey finished undergraduate study (45%), followed by secondary school at around 38%. The majority of respondents are active as entrepreneurs, representing approximately 45%, and students with 34%. Regarding Fintech usage frequency and purpose, only 42 out of 409 people never used Fintech service, compared to 367 who have used Fintech services. This corresponds to the Fintech usage purpose reveal that Indonesian women utilize Fintech services for personal and business objectives with a slight gap of around 5%.

**Table 1:** Respondents’ social and economic characteristics

Characteristic	Criteria	Frequency (*n* = 409)	Percentage (%)
Age	15–25	184	44.99
	26–34	123	30.07
	35–45	77	18.83
	> 46	25	6.11
Education	Elementary school or below	7	1.71
	Junior or senior high school	158	38.63
	Diploma	32	7.82
	Undergraduate	185	45.23
	Master or Doctor	27	6.60
Occupation	Student	140	34.23
	Private employee	48	11.74
	Public employee	28	6.85
	Entrepreneur	177	43.28
	Other jobs	16	3.91
Monthly Income	Less than IDR 3.000.000	171	41.81
	IDR 3.000.000–IDR 5.000.000	135	33.01
	More than IDR 5.000.000–IDR 10.000.000	69	16.87
	More than IDR 10.000.000	34	8.31
Fintech usage frequency (weekly)	Never use	42	10.27
	1 time	107	26.16
	2–3 times	126	30.81
	More than 3 times	134	32.76
Fintech usage purpose	Never use	42	10.27
	Personal	194	47.43
	Business	173	42.30

### Assessing the measurement model

The descriptive statistical analysis and the structural equation model analysis were carried out using the SEMinR package, which has been developed by R core Team (2021) and provide a user-friendly syntax to analyze PLS-SEM in the R statistical environment (Hair et al. 2021). Our research analysis includes two analytic stages: assessing the measurement and structural models.

To establish the reliability and validity of the measurement model, we checked indicator reliability, construct reliability, convergent validity, and discriminant validity. Firstly, we examined the indicator loadings. Table 2 reveals that all indicator loadings of the reflectively measured constructs were well above 0.708, which suggests sufficient levels of indicator reliability (Hair et al. 2019).

**Table 2:** Measurement model results

	Factor loadings	Factor loadings square	VIF	Cronbach’s alpha	Composite reliability (CR)	Average variance extracted (AVE)	rhoA
PU1	0.875	0.765	2.548	0.915	0.940	0.798	0.916
PU2	0.906	0.820	3.245				
PU3	0.892	0.795	2.936				
PU4	0.900	0.810	2.951				
PEU1	0.892	0.796	2.078	0.819	0.892	0.735	0.826
PEU2	0.832	0.692	1.708				
PEU3	0.846	0.716	1.826				
UI1	0.848	0.718	1.873	0.855	0.912	0.775	0.865
UI2	0.890	0.792	2.384				
UI3	0.902	0.814	2.298				
FL1	0.843	0.711	1.822	0.821	0.894	0.737	0.824
FL2	0.837	0.701	1.731				
FL3	0.894	0.800	2.194				
GS1	0.861	0.742	1.879	0.816	0.891	0.732	0.823
GS2	0.894	0.799	2.185				
GS3	0.810	0.655	1.637				
TR1	0.893	0.797	2.279	0.896	0.935	0.828	0.897
TR2	0.919	0.844	3.224				
TR3	0.918	0.843	3.122				
BRI1	0.744	0.554	1.377	0.763	0.863	0.679	0.782
BRI2	0.869	0.755	1.748				
BRI3	0.852	0.726	1.696				
AT1	0.888	0.789	2.368	0.884	0.928	0.811	0.886
AT2	0.918	0.843	2.776				
AT3	0.895	0.802	2.441				
BI1	0.908	0.825	1.558	0.749	0.888	0.799	0.757
BI2	0.879	0.773	1.558				

Secondly, we assessed internal consistency reliability. Joreskog (1971) recommended that higher values of composite reliability indicate higher levels of reliability. Table 2 shows that composite reliability ranged from 0.863 to 0.940, which satisfied recommended value by (Hair et al. 2019). Cronbach’s alpha ranged from 0.749 to 0.915, which was also satisfied with the recommended value. Moreover, another measure of reliability that was used is rohA which is often suggested in between Cronbach’s alpha and the composite reliability (Dijkstra and Henseler 2015). Meeting the above criterion shows that our factor model was reliable. Thirdly, to assess the convergent validity of each construct measure, we calculated the average variance extracted (AVE) of all items on each construct. The acceptable AVE was higher than 0.50, meaning the construct explains 50 percent or more of the indicators’ variance that makes up the construct (Hair et al. 2019). All constructs satisfied the criteria for convergent validity. Fourthly, to assess the discriminant validity, we evaluated Fornel and Larcker’s criterion (1981), which suggested that the square root of the AVEs should exceed the construct correlations (Chin 1998; Fornell and Larcker 1981). Moreover, we also assessed the cross-loadings factors. Tables 3 and 4 show that our measurement model satisfied Fornel & Larcker’s Criterion and good cross-loading factors. From the above analyses, we concluded that our measurement model is reliable and valid.

**Table 3:** Fornel and Larcker criterion

	PU	PEU	UI	FL	GS	TR	BRI	AT	BI
Perceived usefulness (PU)	*0.893*								
Perceived ease of use (PEU)	0.816	*0.857*							
User Innovativeness (UI)	0.304	0.291	*0.880*						
Financial literacy (FL)	0.356	0.296	0.537	*0.859*					
Government support (GS)	0.562	0.609	0.431	0.389	*0.856*				
Trust (TR)	0.577	0.626	0.453	0.401	0.601	*0.910*			
Brand image (BRI)	0.660	0.707	0.410	0.351	0.673	0.653	*0.824*		
Attitude (AT)	0.718	0.682	0.571	0.396	0.673	0.674	0.721	*0.901*	
Behavior intention (BI)	0.664	0.604	0.552	0.427	0.580	0.647	0.685	0.805	*0.894*

FL Criteria table reports square root of AVE on the diagonal (*Italic*) and construct correlations on the lower triangle

**Table 4:** Cross-loading factors

	PU	PEU	UI	FL	GS	TR	BRI	AT	BI
FL1	0.258	0.209	0.454	**0.843**	0.283	0.326	0.249	0.331	0.343
FL2	0.360	0.319	0.439	**0.837**	0.384	0.349	0.363	0.374	0.379
FL3	0.298	0.234	0.488	**0.894**	0.334	0.357	0.292	0.316	0.376
PU1	**0.875**	0.682	0.323	0.402	0.495	0.529	0.553	0.640	0.617
PU2	**0.906**	0.757	0.229	0.292	0.484	0.496	0.583	0.611	0.556
PU3	**0.892**	0.712	0.235	0.317	0.498	0.481	0.581	0.626	0.567
PU4	**0.900**	0.763	0.299	0.263	0.530	0.553	0.637	0.684	0.630
PEU1	0.766	**0.892**	0.237	0.260	0.554	0.558	0.645	0.634	0.558
PEU2	0.644	**0.832**	0.311	0.307	0.509	0.527	0.577	0.557	0.518
PEU3	0.683	**0.846**	0.202	0.193	0.502	0.523	0.593	0.559	0.474
TR1	0.528	0.581	0.454	0.371	0.523	**0.893**	0.589	0.616	0.615
TR2	0.490	0.542	0.412	0.365	0.559	**0.919**	0.564	0.595	0.559
TR3	0.554	0.582	0.368	0.358	0.558	**0.918**	0.626	0.628	0.589
BRI1	0.503	0.547	0.218	0.201	0.381	0.424	**0.744**	0.510	0.466
BRI2	0.578	0.609	0.350	0.298	0.585	0.620	**0.869**	0.615	0.622
BRI3	0.548	0.593	0.424	0.355	0.669	0.550	**0.852**	0.647	0.590
GS1	0.537	0.585	0.370	0.314	**0.861**	0.545	0.662	0.645	0.529
GS2	0.490	0.534	0.383	0.344	**0.894**	0.528	0.569	0.573	0.518
GS3	0.407	0.436	0.354	0.343	**0.810**	0.465	0.489	0.502	0.435
UI1	0.279	0.290	**0.848**	0.422	0.368	0.373	0.357	0.499	0.458
UI2	0.232	0.179	**0.890**	0.446	0.310	0.383	0.324	0.458	0.474
UI3	0.290	0.293	**0.902**	0.538	0.449	0.434	0.397	0.546	0.520
AT1	0.622	0.593	0.543	0.365	0.587	0.610	0.653	**0.888**	0.694
AT2	0.662	0.625	0.538	0.371	0.633	0.635	0.676	**0.918**	0.763
AT3	0.654	0.625	0.464	0.334	0.596	0.576	0.618	**0.895**	0.715
BI1	0.647	0.607	0.466	0.330	0.529	0.618	0.660	0.778	**0.908**
BI2	0.533	0.464	0.526	0.441	0.506	0.534	0.559	0.654	**0.879**

### Assessing the structural model

#### Assess collinearity issues of the structural model

To check potential collinearity issues, we examined the variance inflation factor (VIF) values (Hair et al. 2019). VIF values exceeding 5 indicate potential collinearity issues among the predictor constructs. However, as shown in Table 2, VIF items had a maximum of 3.245, indicating that collinearity problems are not present (Becker et al. 2015).

#### Assess significant and relevant of the structural model relationship

The R^2^ values indicate the explanatory power of the dependent constructs of the research model. In many social sciences, R^2^ values of 75, 50, and 25% can be considered substantial, moderate, and weak explanatory power (Hair et al. 2011). As shown in Table 5, the research model explained 34.6% of the variance in user innovativeness, 66.7% of the variance in perceived usefulness, and 70.4% of the variance in behavioral intention to adopt Fintech. These R^2^ values indicate that our research model had substantial and moderate explanatory power. Perceived usefulness had a significant impact on behavior intention. Perceived ease of use had significant impact on behavior intention and had a significant impact on perceived usefulness. User innovativeness had a significant impact on behavior intention. Financial literacy had insignificant impact on behavior intention, but had a significant impact on user innovativeness. Government supports had insignificant impact on behavior intention, but had a significant impact on user innovativeness. Trust had a significant impact on behavior intention. Brand image had a significant impact on behavior intention. Attitude had a significant impact on behavior intention.

**Table 5:** Results of Path analysis

Hypothesis	Direct Path coefficient	T value	Result
H1: PU—> BI	0.192	3.714^***^	**Accepted**
H2: PEU—> BI	− 0.087	− 1.678*	**Accepted**
H3: PEU—> PU	0.816	42.639^***^	**Accepted**
H4: UI—> BI	0.127	2.853^**^	**Accepted**
H5: FL—> BI	0.042	1.195	Rejected
H6: FL—> UI	0.435	8.966^***^	**Accepted**
H7: GS—> BI	− 0.045	− 1.053	Rejected
H8: GS—> UI	0.262	5.085^***^	**Accepted**
H9: TR—> BI	0.119	2.235^*^	**Accepted**
H10: BRI—> BI	0.171	3.050^**^	**Accepted**
H11: AT—> BI	0.464	8.120^***^	**Accepted**

^***^Represents significant at 0.001 level, ** represents significant at 0.01, * represents significant at 0.05 level

In addition, results from mediating effects (Table 6) reveal that perceived ease of use had a significant indirect impact on behavior intention to adopt Fintech through mediating effect by perceived usefulness. Meanwhile, financial literacy and government support had a significant indirect impact on behavior intention to adopt Fintech through mediating effect of user innovativeness.

**Table 6:** Results of mediating effect

Hypothesis	Mediator	Indirect path coefficient	T value	Result
H3m: PEU—> BI	PU	0.157	3.679	**Accepted**
H6m: FL—> BI	UI	0.055	2.705	**Accepted**
H8m: GS—> BI	UI	0.033	2.394	**Accepted**

#### Moderating effect of saving habit in the COVID-19

A hierarchy PLS-SEM bootstrapping procedure was conducted to assess the influence of Women’s saving habits in the COVID-19 period (WSH) in the relationship between various factors and behavior intention to adopt Fintech. Results from the bootstrapping (Table 7) show saving habits in the COVID-19 time significantly and negatively moderated the relationship between user innovativeness and behavior intention to adopt Fintech. This result refers that higher saving behavior in the COVID-19 period can significantly lower the effect of user innovativeness on behavior intention to adopt Fintech. Meanwhile, there is an insignificantly moderating effect of WSH in the relationship between other variables and behavior intention.

**Table 7:** Summary of moderating effect of saving habit during the COVID-19 period

Hypothesis	Path relationship	Direct path coefficient	R^2^	Result
H12a	PU*WSH—> BI	−0.025	0.709	Rejected
H12b	UI*WSH—> BI	−0.076**	0.715	**Accepted**
H12c	TR*WSH—> BI	−0.042	0.710	Rejected
H12d	BRI*WSH—> BI	−0.048	0.711	Rejected
H12e	AT*WSH—> BI	− 0.032	0.709	Rejected

^**^Represents significant at 0.01 level

To assess the interaction effect in moderation, the study calculated f-Sq effect size (Hair et al. 2022). According to the suggestion from Kenny (2018), the effect size of moderation can be small, medium, and large based on result of f-Sq value 0.005, 0.01 and 0.025, respectively. From our result, f-Sq value of 0.038 indicated that the contribution of the moderating effect on the explanatory power of research model was large.

$$f^{2} = \frac{{R^{2} included - R^{2} excluded}}{{1 - R^{2} included}} = \frac{0.715 - 0.704}{{1 - 0.715}} = 0.038$$

## Discussions and conclusion

The results reveal that almost all investigated variables have a significant relationship with behavior intention to adopt Fintech, excluding government support and financial literacy, which positively impact behavior intention through the mediating effect of user innovativeness.

The most striking result of the study is that perceived ease of use has a significantly negative impact on behavior intention to adopt financial technology services in the case of Indonesian women. This result is inconsistent with the conclusion from the TAM model conducted by Agyei et al. (2020); Chawla and Joshi (2020); Abdul-Halim et al. (2022) and Jain and Chowdhary (2021). This inverse correlation exemplifies that Indonesian female customers may find Fintech applications incomplete or unsophisticated. This phenomenon may be due to the fact that Indonesian women have experience using smartphone apps and may demand more utilities. Moreover, Fintech services in Indonesia could be in an early stage of development that needs more investment to improve and upgrade technologies, including security, ecosystem, and interfaces.

The statistical analyses show that perceived usefulness is still among the important factors in the decision to adopt Fintech among Indonesian women. This result is consistent with most findings from TAM research. As one of the most significant factors, customers have an intention to adopt new technology if they feel this technology would benefit their performance (Singh et al. 2020; Talwar et al. 2020; Rahi et al. 2020). A recent study conducted by Nathan et al. (2022) reveals that perceived usefulness is found to be statistically significant correlated with Fintech adoption.

Although government support and financial literacy have no direct impact on behavior intention to adopt Fintech services, these factors influence user innovativeness. It seems that higher knowledge in finance would stimulate female Fintech users to discover and test new Fintech products. Moreover, Indonesian women tend to be more innovative in Fintech services supported and promoted by governmental policy. The insignificantly results from the impact of government support on behavior intention to adopt Fintech reveal that even though the Indonesia govern involving in the Fintech development during the COVID-19, it seems the support does not yet generate significant results. To accelerate digital financial inclusion, the Indonesian government, through OJK and working with all governors, create the Acceleration of Access to Regional Finance in every province in Indonesia. Related to the reverse impact on financial literacy toward Fintech adoption, it may reveal that in Indonesia, Fintech may promote financial inclusion by allowing people with limited financial literacy to use financial technologies (Nathan et al. 2022).

Trust, attitude and brand image positively impact the behavior intention of Indonesian female customers. Similar findings have also reported by the other previous literatures (Setiawan et al. 2021; Nathan et al. 2022; Tran and Nguyen 2022). This can be implied by Indonesian women perceive Fintech application security and respondents’ convenience assumptions as significant variables affecting Fintech adoption.

Our result from statistical analysis demonstrates that the higher saving behavior during the COVID-19 pandemic reduced the relationship between user innovativeness and behavior intention to adopt Fintech among female customers. This result seems consistent with the functional performance of Fintech providers in Indonesia. COVID-19 could be seen as a motivating force for developing new mobile services. Moreover, COVID-19 leads to higher demand for a loan and a higher risk profile; therefore, some innovative Fintech providers select to reduce their portfolio and aim to provide their services to lower-risk customers with a better income.

In conclusion, understanding the essential variables for adopting Fintech by women in Indonesia is valuable for closing the gender gap in accessing and democratizing financial services for all segments of society. Furthermore, an empirical study conducted by D’espallier et al. (2011) indicates that microfinance disbursement to female customers carries a lower default risk. This phenomenon could also increase the Fintech company’s profitability and potentially accelerate the future of financial inclusion in Indonesia, then promote to achievement of the United Nations Sustainable Development Goals (UN SDGs) related to providing equal financial access to everyone.

## Implications, limitations, and future works

The study revised the efficiency of the TAM model in explaining the intention of women customers to adopt Fintech services. The findings of this article suggest that perceived usefulness, attitude, user innovativeness, brand image, and trust are among the good indicators. Meanwhile, as customers are getting more knowledge in related-technology services, perceived ease of use would negatively impact the decision to adopt new technology and become a barrier. Furthermore, in the context of the COVID-19 pandemic, customers often pay attention to higher saving habits, as the results will impact the relationship between user innovativeness and behavior intention to adopt Fintech services.

The results have important implications for financial firms and technology developers. Fintech companies should improve customers’ attitudes because this factor is the most important factor influencing the decision to adopt Fintech services. In order to achieve a higher attitude from customers’ perspectives, Fintech providers should create more attractive Fintech applications, provide more interesting customer relationships, and broaden their service ecosystem. Moreover, to be successful, Fintech services should be more complete and secure to attain the attention of female customers. This means that they could need to regularly update their applications and provide more secure systems to protect customers’ information and transactions. Moreover, the pursuit of women’s adoption of Fintech must go cohesive with equal participation in the creation and improvement of these financial technologies through incentives for women’s participation in the industry.

Furthermore, this work leads to implications for governments through public policies to achieve higher financial inclusion and reduce the gender gap in access to financial services by promoting the usage of Fintech services. Governments can indirectly influence individual decisions through two channels. First, raising public awareness of financial topics would be essential. Furthermore, the government could provide more training courses to the older and younger generation of women to help them build confidence in approaching financial services. Secondly, the government should improve a transparent policy and sandbox to support new Fintech companies. Receiving technical and administrative assistance from governmental authorities increases the success of Fintech entrepreneurs and improves Fintech’s reputation among users. The COVID-19 situation may reduce the role of customer innovativeness; however, Fintech providers may overcome this problem by creating monetary incentives for using Fintech services.

Although this study provides significant results, some limitations should be mentioned. The explanatory power is 70%, so other important factors should be included in future research. As this is a sample study of a large and diverse population, we accept a certain degree of error. The methodology of empirical analysis and solutions can be adopted in other countries and technological services, considering that the results are affected by the cultural characteristics of the country. For future work, it is also important to conduct a study that examines the drivers and barriers factors for the adoption and use of Fintech, including evaluating the role of Fintech in increasing financial inclusion in Indonesia. Furthermore, the longitude research should investigate how customers’ behavior changes after the COVID-19 pandemic.
